# Realization of a micrometre-scale spin-wave interferometer

**DOI:** 10.1038/srep09873

**Published:** 2015-05-15

**Authors:** O. Rousseau, B. Rana, R. Anami, M. Yamada, K. Miura, S. Ogawa, Y. Otani

**Affiliations:** 1 Center for Emergent Matter Science, RIKEN, 2-1 Hirosawa, Wako 351-0198, Japan; 2 Frontier Research Academy for Young Researchers, Kyushu Institute of Technology, 680-4 Kawazu, Iizuka 820-8502, Japan; 3 Hitachi Ltd., Central Research Laboratory, 1-280 Higashi-koigakubo, Kokubunji-shi,Tokyo 185-8601, Japan; 4 Institute for Solid State Physics, University of Tokyo, Kashiwa 277-8581, Japan

## Abstract

The recent development of spin dynamics opens perspectives for various applications based on spin waves, including logic devices. The first important step in the realization of spin-wave-based logics is the manipulation of spin-wave interference. Here, we present the experimental realization of a micrometre-scale spin-wave interferometer consisting of two parallel spin-wave waveguides. The spin waves propagate through the waveguides and the superposition or interference of the electrical signals corresponding to the spin waves is measured. A direct current flowing through a metal wire underneath one of the spin-wave waveguides affects the propagation properties of the corresponding spin wave. The signal of constructive or destructive interference depends on the magnitude and direction of the applied direct current. Thus, the present work demonstrates a unique manipulation of spin-wave interference.

Many new concepts of information transport using spin waves have been proposed in recent years^1^,^2^,^3^,^4^,^5^,^6^,^7^,^8^,^9^,^10^. Spin waves can be regarded as the collective spin resonance of electrons in a magnetic material. Interestingly, one can excite spin waves locally and detect them after propagation^3^,^10^,^11^,^12^,^13^. These propagating spin waves carry angular momentum like diffusive spin currents in spintronics. Because they are waves, one can use the phase to convey information. Indeed the propagation length of such waves is notably longer than the electron spin diffusion length in semiconductors or metals. Spin waves can thus be applied to long-range propagation of information, as the loss of information per unit propagation length is smaller than that of diffusive spin currents at room temperature.

Spin waves can be generated in various ways^3^,^14^,^15^ and amplified^16^. Recent reports have shown the possibility of switching the propagation direction at will^17^,^18^. The first important step in the development of spin-wave-based devices would be the realization of spin-wave logics. The basic building block of spin-wave logics is the spin-wave interferometer where the interference of two or more spin waves can be manipulated. However, spin-wave-based logics are mainly proposed theoretically down to nanometre scale^6^,^19^. Spin-wave interferences have been reported experimentally in micro stripes using rising and falling edges of electrical pulses^20^,^21^ and oppositely propagating spin waves^22^ that do not allow free manipulation of the interference. However, there have been very few reports on Mach–Zehnder-type spin-wave interferometers on millimetre scale with yttrium iron garnet as a magnetic medium^14^,^23^.

Herein we demonstrate the manipulation of spin-wave interference using a Mach–Zehnder-type interferometer on a micrometre scale. Like other published results on spin-wave propagation and spin-wave logic gates^12^,^14^,^17^, it is a step towards spin-wave-based logics on micrometre and nanometre scales. In the case of our interferometer, the spin wave propagating through one branch has a shifted dispersion curve compared with the spin wave in the other branch, resulting in a phase difference between them.

## Results

### Sample characteristics and concept

**Figure 1:**
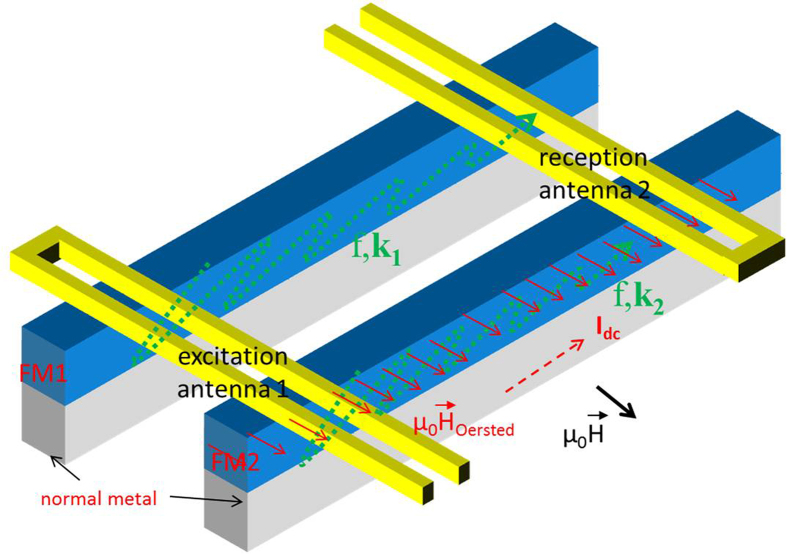
Sample layout. Sketch of the device layout and operating principle of the spin-wave interferometer with two spin-wave waveguides.

We measure interference signals on various devices. Figure 1 is a sketch of the device layout along with the operating principle. In the present work, under a fixed applied magnetic field (biased field) (**H**), a continuous radio-frequency (rf) current (*I*_*rf*_) is passed through antenna 1 at frequency (*f*) to generate an rf magnetic field. This rf magnetic field generates spin waves at the same frequency *f* in two waveguides (FM1 and FM2) of Co_20_Fe_60_B_20_ above a normal metal, especially under the resonance condition. The resonance condition depends on **H**, *f*, and the wave vector (**k**). Spin waves propagate through the two spin-wave waveguides FM1 and FM2. Direct current (*I*_*dc*_) can also be applied through FM2 to change the propagation properties of the spin wave propagating through it. The electrical signal produced inductively by FM1 interferes with that produced by FM2 within antenna 2, where the total interference signal is measured. In the following, we show that the measured interference signal entirely depends on the amplitudes of and relative phase between the spin waves. Thus, even though the spin waves do not actually interfere themselves, we use the term *spin-wave interference* in the following to remind us that the properties of spin waves are controlled for the realisation of interference. The measurements are carried out with a vector network analyser (VNA). Each antenna consists of two parallel branches or arms with width of 4 μm and edge-to-edge separation of 4 μm. The centre-to-centre separation between the two antennas is of the order of 20 μm. The antennas are electrically isolated from the waveguides by a layer of Al_2_O_3_ that is 100 nm thick. The spin-wave waveguides have a width of either 20 or 10 μm and length of 250 μm to avoid spin-wave confinement from the edges along their lengths^24^,^25^. To avoid the detection of a spin wave reflected from the edges along the lengths, the antennas are placed at the centre of the waveguides, perpendicular to the length. The centre-to-centre distance between the two waveguides is chosen as 40 μm to minimize dynamic dipolar coupling. **H** is applied in plane and perpendicular to the spin-wave waveguides (see also Methods). In the following, we concentrate on ferromagnetic waveguides that are 20 μm wide and 20 nm thick with a propagation distance of 20 μm before detection and an applied magnetic field of *μ*_*0*_*H* = 75 mT. A comparison with other devices is made later in the article.

**Figure 2:**
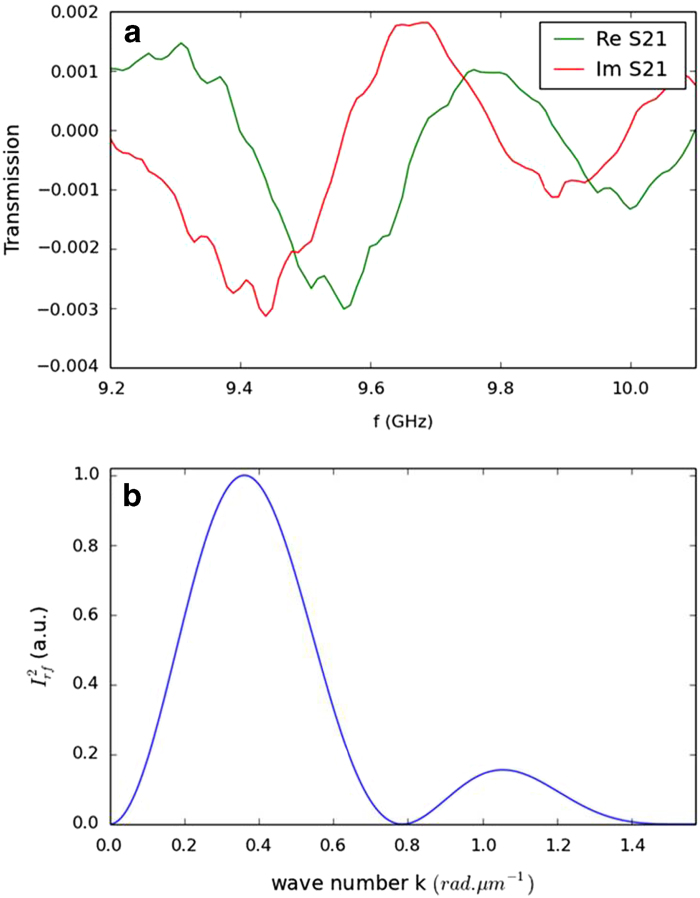
Transmitted signal and efficiency of the antenna in excitation of the wave vector. (**a**) A typical measurement of the transmission versus the frequency after 20 μm of propagation in 20-μm-wide, 20-nm-thick CoFeB waveguides. The applied magnetic field is *μ_0_H* = 75 mT. No direct current is applied through FM2. (**b**) Normalized Fourier transformation of the power delivered by our antennas (I_2_^rf^ ) versus the wave vector (**k**) of the spin wave.

From the measurement of the reflected spin-wave signal (S11), we obtained a resonance frequency of 9.6 GHz with a **k** of 0.4 rad.μm^−1^. This is in agreement with the resonance frequency calculated using the analytical formula for the dispersion relation^26^,^27^ given by 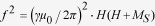

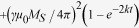
, with *γ*/2*π* = 27.5 GHz/T, *μ*_*0*_*M*_*S*_ = 1.45 T, and *t* being the thickness of the ferromagnetic layer. A typical transmitted signal (S21), from antenna 1 to antenna 2, measured with the VNA at *I*_*dc*_ = 0 is presented in Fig. 2(a). The Gilbert damping α is determined as 0.9 × 10^−3^ ± 0.1 × 10^−3^. The propagation length is estimated as 11 μm (see also Supplementary Information). Oscillations in the transmitted signals, in phase (Re S21) and out of phase (Im S21) with the excitation signal, are observed. The oscillations in the transmitted signals are the result of exciting several **k** of propagation with the antenna^10^,^11^,^13^. Indeed, the geometry of our antennas allows us to excite and detect several **k** as presented in Fig. 2(b). The excited **k** vectors are a continuous peak centred at *k* = 0.4 rad.μm^−1^ (a secondary peak is not observed), which corresponds to the wavelength of 16 μm imposed by the geometry of the antennas. The width of the spectrum of the **k** vector and the damping α together contribute to the width of the spin-wave spectra. A change in the resonant **k** arising from the dispersion relation affects the phase of the spin wave after propagation and thus leads to oscillations of the transmission signals (see also Supplementary Information). In the case of our interferometer, for a fixed *f* and **H**, *I*_*dc*_ applied through FM2 generates an Oersted field that affects the effective magnetic field of FM2. *I*_*dc*_ also generates Joule heating that reduces *M*_*S*_. Both the Joule heating and Oersted field contribute to the modification of the wave vector value *k* from *k*_*1*_ (as in FM1 without *I*_*dc*_) to *k*_*2*_ (as in FM2 with *I*_*dc*_). Thus, the spin waves in the two spin-wave waveguides have different phases when they arrive at the detection antenna. It is worth noting that a change in the **k**, effective field and/or *M*_*S*_ affects the amplitude of the spin wave. The Oersted field is expected to reach 2.0 mT for a *I*_*dc*_ of 90 mA, while the measured value is about 2.1 mT. This corresponds to a change in resonance frequency of 130 MHz, while a change of ~250 MHz in resonance frequency is needed to change the phase of the spin wave by 180°. Thus, the Oersted field itself is not strong enough to notably affect the spectrum in Fig. 2(a) to obtain destructive interferences. The increase in temperature due to Joule heating is expected to be around 250–300 °C when a high current density (~1 × 10^11^ A.m^−2^) is applied to achieve destructive interference. This would also induce a change in *M*_*S*_ which increases the phase of the spin wave by 180°. This leads to the detection of spin-wave generated interferences by antenna 2 as we demonstrate below.

### Experimental demonstration of the manipulation of spin-wave interference

**Figure 3:**
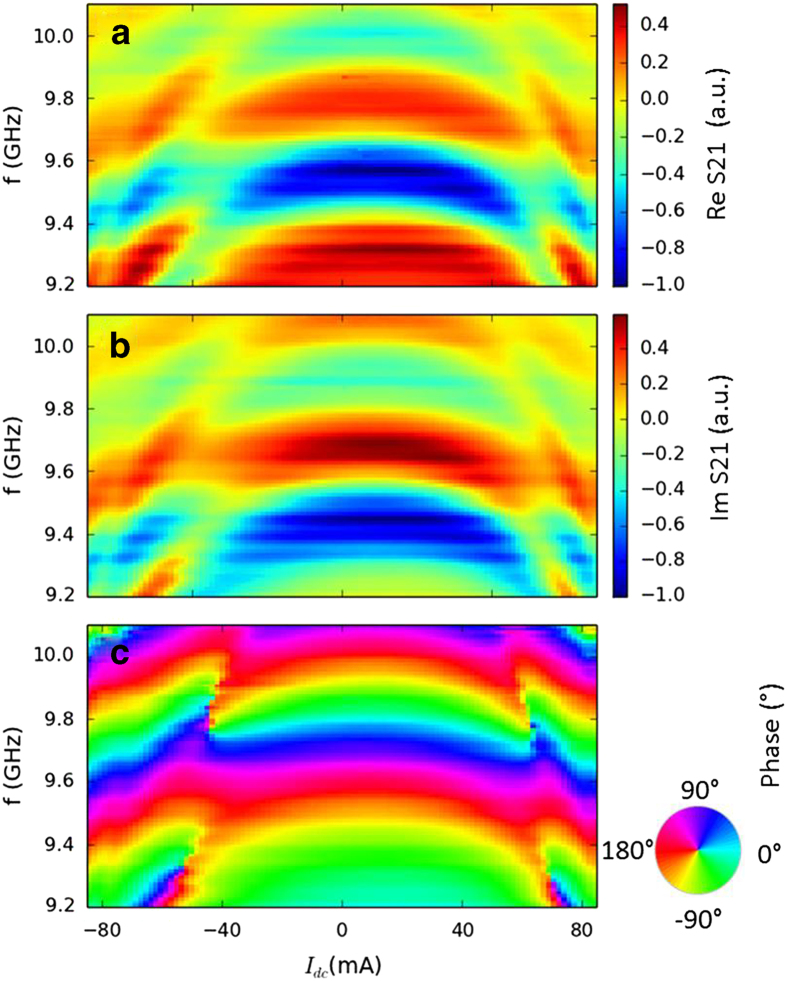
Maps of interferences at 75 mT. The sample is the same as that described in Fig. 2(a). The real part of the transmission between antennas 1 and 2 (Re S21) for different frequencies and applied I_dc_ are shown in (**a**) and the imaginary part (Im S21) in (**b**). The phase because of spin-wave transmission detected by antenna 2 is presented in (**c**).

To evaluate the efficiency of the interferometer, we measured the transmitted spin-wave signals with a VNA at different *f* and applied *I*_*dc*_. The *f* is varied from 9.2 to 10.1 GHz in steps of 10 MHz. For each *f*, *I*_*dc*_ sweeps between −85 and 85 mA. Figures 3(a) and (b) show colour maps of the measured Re S21 and Im S21 signals as functions of *I*_*dc*_ and *f* under a bias magnetic field *μ*_*0*_*H* = 75 mT. The maps have three important features. First, Re S21 (Fig. 3(a)) and Im S21 (Fig. 3(b)) evolve with *I*_*dc*_ in a similar way but with a phase difference of 90°. This is evidence that the spin wave is transmitted. The second feature is the near-perfect symmetry of the maps with respect to the sign of *I*_*dc*_. This can be understood as follows. The Oersted field is added to the bias magnetic field for the determination of the resonant condition. However, this addition depends on the sign of *I*_*dc*_. Thus, for example, when Re S21 is zero for a given frequency without *I*_*dc*_, one would obtain either a positive or negative Re S21 while applying a positive or negative *I*_*dc*_, respectively. The transmitted signal should then have predominantly asymmetric behaviour with respect to the sign of *I*_*dc*_ if there is only an Oersted field. However, negligible asymmetry is observed here for real and imaginary components of the transmitted signal at each frequency. Because the contribution from Joule heating (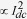) is the same for positive and negative *I*_*dc*_, it tends to generate symmetric curves irrespective of the sign of *I*_*dc*_. Thus, the symmetry in our maps is mainly a consequence of Joule heating and the small asymmetric behaviour is a consequence of the Oersted field. Thus, the effect of the Oersted field on the dispersion is secondary to the effect of Joule heating of the sample under a bias magnetic field of 75 mT, and for *I*_*dc*_ is sufficiently strong to noticeably affect the transmitted signals. The dominant nature of Joule heating on the modification of the dispersion (resonance) curve is in good agreement with measurements (see Supplementary Information) realised with only one spin-wave waveguide through which *I*_*dc*_ flows. The third feature is that the sign of the transmitted signal barely changes with *I*_*dc*_. As the spin wave in FM1 is unaffected by *I*_*dc*_, it creates a baseline around which the transmitted signal oscillates with *I*_*dc*_.

**Figure 4:**
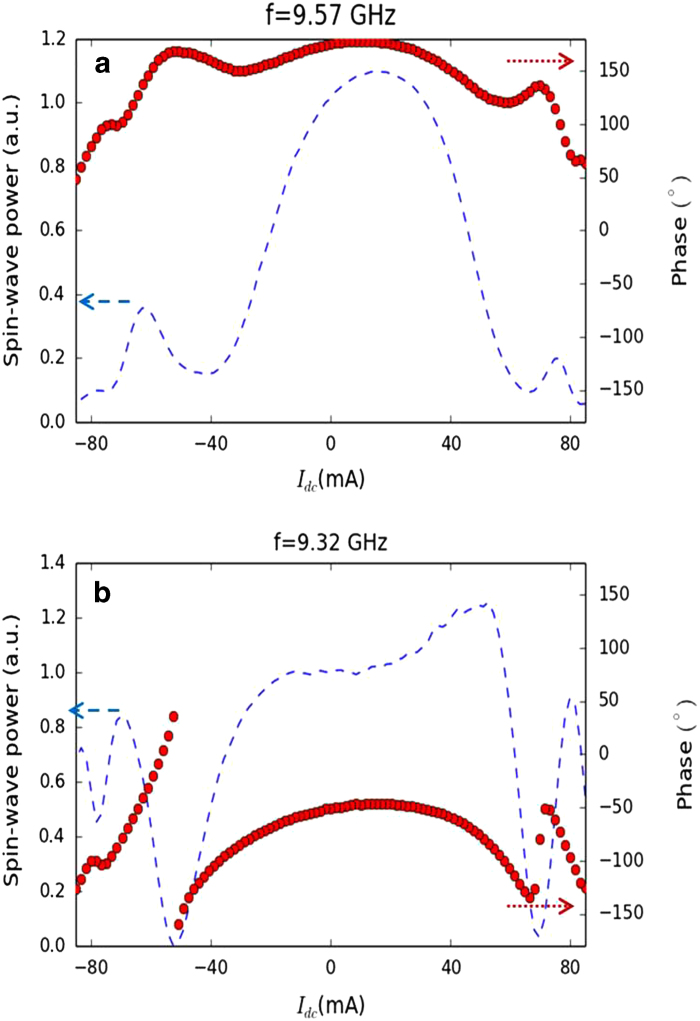
Constructive and destructive interference. Transmitted power (blue dashed line) and corresponding phase (red circular points) of the spin wave versus the applied current (*I*_*dc*_) are shown for frequencies of 9.57 and 9.32 GHz, respectively in (**a**) and (**b**).

Using Re S21 and Im S21, we calculate the phase of the spin-wave signal detected by antenna 2. As expected, the resulting phase map (Fig. 3(c)) has the same global behaviour as the transmission maps. However, in the phase map, there are four critical points around which the phase can take any possible value in response to a small change in *I*_*dc*_ or the applied frequency. The four points are at (9.83 GHz, −45 mA), (9.79 GHz, 61 mA), (9.32 GHz, −52 mA), and (9.27 GHz, 68 mA). To understand this behaviour, we present the transmitted power and corresponding phase for two representative frequencies, 9.57 GHz (Fig. 4(a)) and 9.32 GHz (Fig. 4(b)). The phase varies smoothly with *I*_*dc*_ for *f* = 9.57 GHz, but abruptly for *f* = 9.32 GHz at *I*_*dc*_ = −52 mA. Additionally, in the latter case of *f* = 9.32 GHz, the transmitted power reaches zero at *I*_*dc*_ = −52 mA. Therefore, power associated with the spin-wave propagation also reaches zero. This means that the two spin waves in FM1 and FM2 have opposite phases but equal amplitudes, corresponding to totally destructive interference. This also explains the lack of continuity in the phase (for example, the phase suddenly jumps from −161° to 36° at 9.32 GHz and −52 mA) at the four critical points. Clearly, we cannot define a phase if there is no measurable signal. A consequence is that a slight change in the frequency or current can affect the amplitude and phase of the interference signal. In the case of positive current, a slightly higher current is required to tune **k** for totally destructive interference at the same frequency because of the competition between the Oersted field and Joule heating. However, the higher current also results in a increment of temperature (via Joule heating). The spin-wave amplitude thus differs from that in the case of negative current. Therefore, a small signal is still transferred and the phase becomes continuous. In this case, the interference is partially destructive. There is totally destructive interference at 9.27 GHz and 68 mA for positive current, against 9.32 GHz and −52 mA for negative current. For frequencies far from frequencies of totally destructive interference (e.g., 9.57 GHz), the amplitude of the spin wave in FM2 is too different from that in FM1 for there to be totally destructive interference, even when the phases are opposite. Thus, in spite of a notable decrease in the received power (which is sufficient for logic devices) arising from partially destructive interference, the phase is continuous with smaller variations than in the case of totally destructive interference.

We note here that no other effects of the direct current on the spin wave, such as a spin-wave Doppler shift^28^ were observed for our samples (see Supplementary Information for more details). Thus, at fixed frequency, *I*_*dc*_ only affects the spin-wave dispersion relation through Joule heating and the Oersted field. Both contribute to the manipulation of the interference by modifying the **k** of the spin wave propagating through FM2. It is worth noting that the received signal is a wave and not simply a pulse^14^. Therefore, the signal can be reused especially after amplification^16^ in more complex spin-wave devices. We can change the number of oscillations or periods of the transmitted signal at *I*_*dc*_ = 0, as seen in Fig. 2(a) by changing either the distance between emission and reception^11^,^13^ antennae or the thickness of the ferromagnetic layer. In this case, a different value of *I*_*dc*_ is required to obtain the required change in phase of 180° in one arm of the interferometer compared with the other arm to achieve destructive interference. However, the physics remains the same.

**Figure 5:**
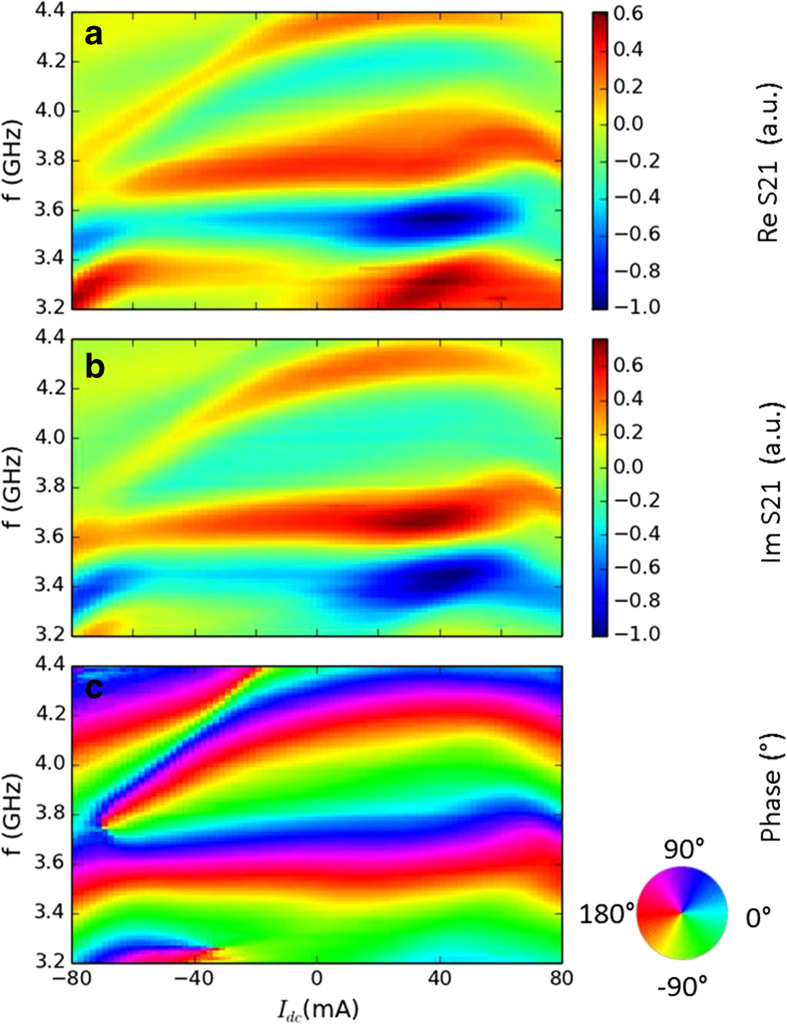
Map of interference under a lower-bias magnetic field of 10 mT. The sample is similar to that described in Fig. 2 with 10 nm-thick CoFeB. Interference maps similar to those in Fig. 3 are presented in (**a**), (**b**) and (**c**) for a bias magnetic field of 10 mT and propagation distance of 19 μm.

The application of a stronger magnetic field to any device could be problematic. Thus, last, we present the interference obtained under a lower-bias magnetic field of 10 mT. The colour map of the interference as a function of frequency and current is presented in Fig. 5. Unlike the case for a stronger magnetic field, the map is mainly asymmetric with respect to the sign of *I*_*dc*_. As explained above, this means that the Oersted field dominates the variations in the dispersion relation. The reason is explained as follows. For a weaker magnetic field, the Oersted field leads to greater relative variations in the effective magnetic field. However, we reach totally destructive interference at a frequency around 3.75 GHz and *I*_*dc*_ around −70 mA, where the phase is not well defined. The predominance of the Oersted field over Joule heating means that the colour map of interference is less symmetric with respect to the sign of *I*_*dc*_. We can define a threshold current here. Above this threshold current, an increase in positive current modifies the transmission more through Joule heating (where the effect is proportional to *I*_*dc*_^2^) than through the Oersted field (where the effect is proportional to *I*_*dc*_). This threshold current is around 20–25 mA in Fig. 3. From the dispersion relationship, one would expect the threshold current to be around 45–56 mA in Fig. 5, where it is in fact found to be around 45–50 mA. This good agreement confirms the relative effects of the two contributions. However, the important point is that interference can also be driven from constructive (no current) to destructive under a weak applied magnetic field. A weak magnetic field can be applied by dipolar coupling with a permanent magnet. This consideration has to be taken into account when determining the magnetic field required for spin-wave-based devices in spintronics.

## Discussion

We experimentally demonstrated how spin-wave interference can be manipulated with a spin-wave interferometer on the micrometre scale. Unlike previous demonstrations on the millimetre scale^14^,^23^, the entire length of the interferometer is used to produce interference. Here, we used direct current to modify dynamically the propagation properties of the spin wave in one of the two branches of our interferometer. We make the most of both Joule heating and the Oersted field produced by the direct current to control the wave vector and hence the phase of the propagating spin wave in one branch compared with that in the other branch for the same excitation frequency. The advantage of controlling the properties of spin-wave propagation with direct current is the ability to freely manipulate the interference. However, to reduce energy consumption, interference can also be manipulated by tuning the resonant conditions of the two arms of the interferometer either by choosing a different width (at smaller width, the width of the spin-wave waveguide also affects the resonant condition) or by choosing different ferromagnetic materials or magnonic crystals^29^ for each spin-wave waveguide. These measurements can be extended in the time domain with modulated pulse excitation as in the literature^14^. Last, we used two antennas in our experiment (one for excitation of the spin wave in both waveguides and another for detection of the spin wave from both waveguides after propagation). However, one can also use one antenna (or any other instrument for magnon injection with a controllable **k** vector) per spin-wave waveguide with controlled phase (as in references 22 and 23) for excitation and one global antenna for detection. This would lead to spin-wave-based logics. If we have two antennas for the injection and still one for the reception, the two inputs would need to be out of phase to receive a signal at the receptor in the case of destructive interference. This would correspond to a kind of XNOR gate. With interferometers that are more complex (e.g., interferometers with more spin-wave waveguides), other logic functions can be designed, especially if the spin-wave-generated signal is stronger than the baseline^12^ and the spin-wave propagation direction is imposed^17^.

## Methods

### Sample preparation

The samples were prepared in four steps. First, a Co_20_Fe_60_B_20_ (20 or 10 nm)/Ta(5 nm)/Ru(10 nm)/Ta(5 nm) layer was deposited on a SiO_2_/Si wafer by sputtering. Second, the spin-wave waveguides were defined by optical lithography and etching with an argon ion beam. Third, the 100 nm thick insulator Al_2_O_3_ was deposited by sputtering everywhere except at the extremities of the spin-wave waveguides. Finally, the gold antennas Ti(4 nm)/Au(100 nm) and direct-current contacts were deposited using a combination of optical lithography, electron beam evaporation and lift-off processes.

### Measurement

The measurements were made with a fixed in-plane bias magnetic field applied perpendicular to the length of the spin-wave waveguide with a magnetic field excitation generated by rf current, while sweeping the direct current applied through FM2. In spite of the good signal/baseline ratio obtained in a previous study^12^, we preferred to make measurements with a VNA. Thus, to remove the signal corresponding to inductive coupling between the two antennas from the transmitted spin-wave signals, measurements were performed under two magnetic fields, the one of interest and a reference magnetic field of 170 mT, where no spin waves were detected in the region of the measurement frequency. We then numerically removed the signals measured under the reference field from the signals measured under the field of interest to extract the signals corresponding only to the spin-wave propagation. The VNA basically measured two components of the transmitted signal: the real component (Re S21) that is in phase with the excitation signal, and the imaginary component (Im S21) that is out of phase with the excitation signal. The signal corresponding to spin-wave propagation was thus clearly determined along with its phase.

## Author Contributions

O.R. conceived the project. O.R., B.R., K.M., M.Y. and R.A. prepared the samples. O.R., B.R. and R.A. performed the measurements. O.R., B.R., R.A., S.O. and Y.O. discussed the results. O.R. wrote the manuscript with input from B.R., S.O., and Y.O. Y.O. supervised the project.

## Additional Information

**How to cite this article**: Rousseau, O. *et al.* Realization of a micrometre-scale spin-wave interferometer. *Sci. Rep.* **5**, 9873; doi: 10.1038/srep09873 (2015).

## Supplementary Material

Supplementary Information
