# Supplementary material for: Realization of a micrometre-scale spin-wave interferometer
Source: Sci Rep. 2015 May 15;5:9873. doi: 10.1038/srep09873 (PMC4432312; doi:10.1038/srep09873)
Supplement: Supplementary Information [file srep09873-s1.pdf]

Supplementary information

Realization of a micrometre-scale spin-wave interferometer

O. Rousseau[1], B. Rana[1], R. Anami[1,2], M. Yamada[3], K. Miura[3], S. Ogawa[3], and Y. Otani[1,4]*

[1)] Center for Emergent Matter Science, RIKEN, 2-1 Hirosawa, Wako 351-0198, Japan
[2)] Frontier Research Academy for Young Researchers, Kyushu Institute of Technology, 680-4 Kawazu, Iizuka 820-8502, Japan
[3)] Hitachi Ltd., Central Research Laboratory, 1-280 Higashi-koigakubo, Kokubunji-shi, Tokyo 185-8601, Japan
[4)] Institute for Solid State Physics, University of Tokyo, Kashiwa 277-8581, Japan

*Email: yotani@riken.jp

1. Oscillations in the transmitted signal

The presence of oscillations is known to be a sign of transmission. One can study it to extract the propagation velocity of spin-wave. As we have explained in the article that at a fixed bias magnetic field (**H**), an increasing *f* decreases the wave vector (**k**). The phase delay (ϕ) of the received spin-wave is also changed accordingly. This is the consequence of the spin-wave dispersion relation given by $\omega^2 = \omega_0(\omega_0 + \omega_M) + \frac{\omega_M^2}{4}(1 - e^{-2kt})$. A phase change of 2π in the transmitted spin wave corresponds to a change in k of $\delta k = 2\pi/D$, where D is the centre-to-centre separation of two antennas. Thus the group velocity (v_g) can be expressed as $v_g = \delta\omega/\delta k = \delta f \cdot D$, where $\delta f/4$ is the quarter of the period of the oscillations obtained from the Fig. 2. In our case, v_g corresponds to 6.4 ± 0.3 km/s. Considering damping constant (α) = $0.9 \times 10^{-3} \pm 0.1 \times 10^{-3}$, the propagation length (= $v_g/\alpha\omega$) can be estimated as 11 µm. The value of v_g determined from experiment is in good agreement with the v_g determined theoretically by using: $v_g = \frac{d\omega}{dk} = \frac{\omega_M^2 t}{4\omega} e^{-2kt}$, which gives v_g = 5.5 km/s. The agreement is similar to the one reported in references 10, 11 or 13. We agree with reference 13 which suggests that the small discrepancy between experimentally observed value and theoretically calculated value of v_g is related to the size of the antenna compared to the spin wave propagation length.

2. DC current and spin wave transmission

To study the effect of the dc currents on the spin-wave behaviour, we investigated single ferromagnetic waveguide of 20 µm wide and 20 nm thick CoFeB. The centre-to-centre distance between two antennas was 19 µm. The antennas are the same as in the main part of the article. A bias magnetic field of 75 mT was applied in plane of the waveguide and perpendicular to its length.

2.1 Effect of Joule heating and Oersted field

In Fig. S1, we present the absorption of spin-wave power for 3 different dc currents: +90 mA, –90mA and 0 mA. As it is absorption, peaks are negative for the bias magnetic field of 75 mT. If there is the only contribution from Oersted field, the peak corresponding to 0 mA current should be in between the peaks corresponding to +90 mA and –90 mA. However, this is not at all the case here. The frequency difference between high current (average of the resonance frequency between +90 mA & –90 mA) and zero current is even bigger than the frequency difference between positive (+90 mA) and negative (–90mA) current. The other parameter of the dispersion relation which can be influenced by dc current is the saturation magnetization via the Joule heating. This dominates the drift in frequency while a dc current is applied. The asymmetry in transmitted signals between positive and negative dc current is the consequence of the Oersted field which is estimated as 2.1 mT. From the calculation we expect the Oersted field to be around 2.0 mT. These two values are in good agreement. The global shift of the resonance peak at 90 mA dc current (average value of the resonance peak between +90 mA and –90 mA) from the resonance peak at 0 mA corresponds to a change in $\mu_0 M_S$ from 1.45 T to 1.20 T. From reference 30, it corresponds to an increase in temperature of about 250-300°C. We also measured the resistance of the spin-wave waveguide as a function of dc current. This also gives us an estimation of the increase in temperature of about 200-300°C. Again here the agreement is correct.

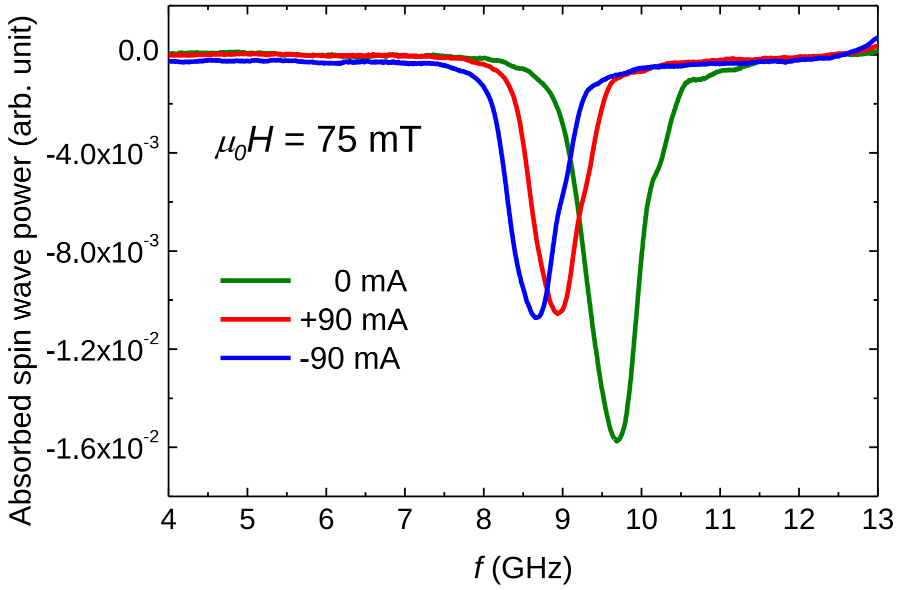

Figure S1. Influence of the dc current on the ferromagnetic resonance.

2.2 **Spin Hall effect**

The metal under our ferromagnetic is Ta. In a particular phase, the Ta has a strong spin-orbit coupling. Thus a dc current flowing through it generates a dc spin-current which can modify the amplitude of the spin wave[31]. This dc spin current will act as a pumping term, thus it will generate a difference in peak power of spin wave between positive and negative dc charge currents. As it can be seen in the Fig. S2, the amplitudes of the two peaks are the same. It means no spin Hall effect is observed here. Our Ta should not be in the particular phase where it can show significant spin Hall effect. Again, most of the current flows through Ru and the ferromagnetic metal. The two effects reduce the spin current given by the Ta. Even if there is spin Hall pumping from an adjacent layer, only the asymmetry in the transmitted signal between positive and negative current will be enhanced, but the interferometer would still be operational.

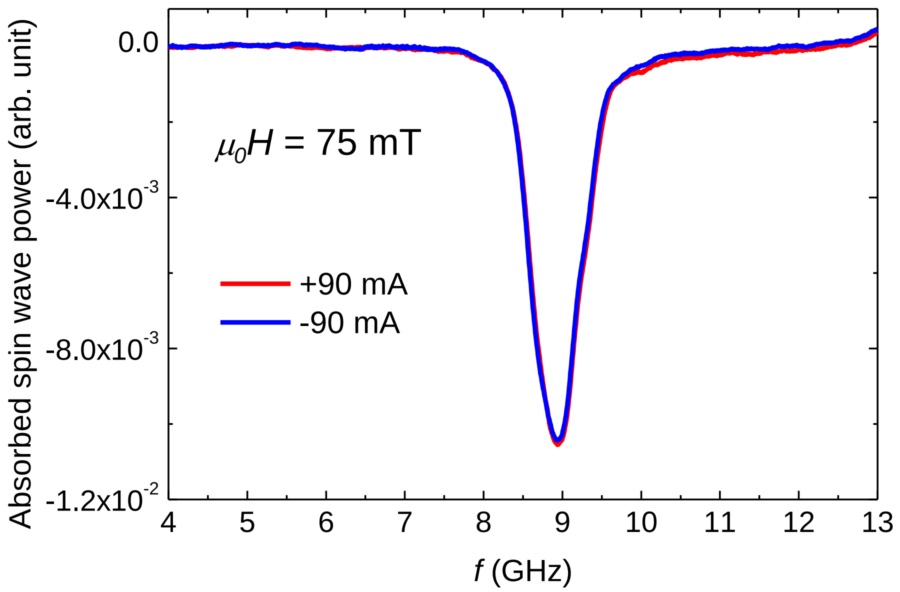

Figure S2. The curves correspond to the same curves of figure S1 with a dc current of +90 mA and –90 mA. This time we shift the frequency of the curve corresponds to –90 mA in order to compare the difference of absorption power between the spin waves at two different currents.

2.3 Doppler effect

As pointed above, some dc current also flows through the ferromagnetic. It has been demonstrated that the spin torque generated by the spin-wave polarization of this current can induce Doppler effect[28]. This Doppler effect will modify the frequency of propagation, making a frequency difference between the spin-waves propagating in opposite direction[28]. In our case (as shown in Fig. S3), the spin-waves propagating in opposite directions have the same phase or resonance frequency. Thus the spin-waves are not sensitive to the Doppler effect in the range of our measurements. The current density inside the ferromagnetic layer should be higher to obtain a measurable Doppler effect. This would happen in narrower stripes where higher current density can be reached. However, in that case Doppler effect will only change the values of dc current for destructive interference, but the interferometer would still have the same global behavior as now. The reason for the differences of amplitude is well known and explained in reference 5, 11 and the references thereby.

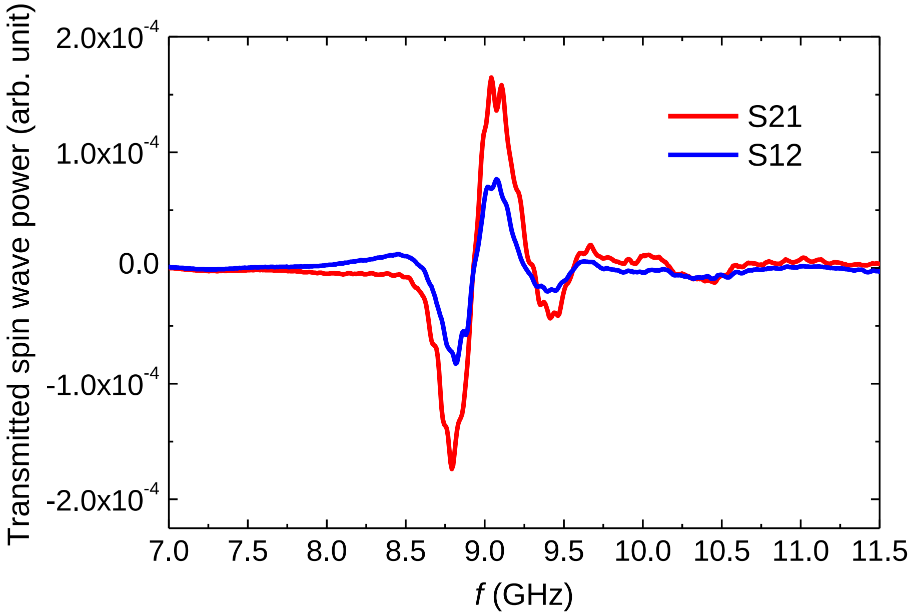

Figure S3. The real part of transmitted spin wave signal from antenna 1 to antenna 2 *i.e.* S21 and in the reverse direction *i.e.* S12 are shown. The applied dc current is +90 mA.

Thus we demonstrate that the spin wave amplitude (absorption is spin-wave amplitude before propagation) at a given frequency is shifted because of combined effect of Oersted field and Joule heating. As explained in the article, a shift in frequency makes a shift in **k** vector at fixed frequency and thus the phase of the received spin wave.

Supplementary References

30. Zych, W. & Milczarek, J. J. Magnetic properties of $Fe_{80-x}TM_xB_{20}$ and $Fe_{80}Mn_xSi_{12}B_8$ amorphous alloys. *Phys. Status Solidi (a)* **90**, K165 (1985).

31. An, K. *et al.* Control of propagating spin waves via spin transfer torque in a metallic bilayer waveguide. *Phys. Rev. B*, **89**, 140405 (2014).
